# Elevated serum triglyceride predicts recurrence of colorectal polyps in patients with advanced adenomas

**DOI:** 10.1186/s12944-020-01388-3

**Published:** 2020-09-23

**Authors:** Boying Liu, Pingwu Wen, Xiaodong Gu, Ruiqiang Weng, Sudong Liu

**Affiliations:** 1 Department of Gastroenterology, Meizhou People’s Hospital (Huangtang Hospital), Meizhou Hospital Affiliated to Sun Yat-sen University, No. 63 Huangtang Road, Meijiang District, Meizhou, 514031 P. R. China; 2 Research Experimental Center, Meizhou People’s Hospital (Huangtang Hospital), Meizhou Hospital Affiliated to Sun Yat-sen University, No. 63 Huangtang Road, Meijiang District, Meizhou, 514031 P. R. China; 3 Guangdong Provincial Key Laboratory of Precision Medicine and Clinical Translational Research of Hakka Population, No. 63 Huangtang Road, Meijiang District, Meizhou, 514031 P. R. China

**Keywords:** Colorectal polyp, Recurrence, Lipid profile, Triglyceride, Risk factor

## Abstract

**Background:**

Recurrence of colorectal polyps is common and impacted by various factors. This study was performed to explore the association between lipid profiles and recurrence of colorectal polyps.

**Methods:**

This study retrospectively analyzed the lipid profiles of 435 patients who underwent colonoscopy with removal of colorectal polyps and assessed recurrence of polyps by follow-up colonoscopy. Multivariate regression logistic analysis was used to evaluate the association between lipid profiles and polyp recurrence.

**Results:**

During the 1.5-year follow-up, recurrence of colorectal polyps was observed in 135 of 435 patients (30.34%). Patients with recurrent polyps showed a higher level of triglycerides (*P* = 0.006) and lower levels of high-density lipoprotein cholesterol (*P* = 0.008) and apolipoprotein A1 (*P* = 0.033). The multivariate regression logistic model suggested that an elevated triglyceride level was an independent risk factor for polyp recurrence (odds ratio, 1.55; 95% confidence interval, 1.02–2.35; *P* = 0.039) in patients with advanced adenoma.

**Conclusions:**

Lipid profiles are associated with recurrence of colorectal polyps. An elevated triglyceride level is an independent risk predictor of polyp recurrence in patients with advanced adenoma.

## Introduction

Colorectal cancer (CRC) is the second and third most common cancer in women and men, respectively, worldwide. It is also the fourth most deadly cancer worldwide [1]. In total, 1.8 million persons were reportedly diagnosed with CRC in 2018, resulting in the death of about 881,000 of these patients [2]. Endoscopic resection of colorectal polyps has effectively reduced the CRC-related mortality rate [3]. However, recurrence is common and remains a major concern for patients after resection [4–6]. Therefore, identification of risk factors associated with polyp recurrence is needed to improve the outcomes of patients with polyps and reduce the incidence of CRC.

CRC is known to develop from colorectal polyps, which are lesions that grow on the mucosal surface and protrude into the colorectal lumen [7]. According to the World Health Organization, colorectal polyps are divided into four types: adenomatous, inflammatory, hyperplastic, and hamartomic polyps [8]. The occurrence rates of different types of polyps widely range from 1 to 43% [6]. Specific factors have been reported to contribute to polyp recurrence, such as age, sex, lifestyle, and polyp characteristics [9]. Obesity and the body mass index (BMI) have long been studied as risk factors for polyp recurrence [10–12]. Researchers have found that obesity promotes the expression of insulin and insulin-like growth factor-1 (IGF-1), causing the transformation of non-advanced colorectal polyps to advanced polyps and triggering recurrence [13]. Several recent studies have shown that metabolic syndrome is associated with an increased risk of recurrent colorectal adenoma [14, 15]. Metabolic factors (age, BMI, and fasting blood glucose) significantly accelerate the development of recurrence after removal of adenomas [15].

The relationship between lipid profiles and colorectal polyps has been extensively studied. Some studies have shown that the serum triglyceride (TG) and cholesterol levels are associated with an increased risk of colorectal adenoma [16, 17], while other studies have failed to confirm such a link or have suggested an inverse relationship between serum lipid levels and colorectal adenoma [18, 19]. A recent study revealed that increased serum TG and low-density lipoprotein cholesterol (LDL-C) levels promote the formation of colorectal polyps [20]. The underlying mechanisms have not yet been fully elucidated, but two pathways might be involved. One suggests that lipid abnormalities are involved in the development of hyperinsulinemia and insulin resistance, which inhibit apoptosis by interacting with the IGF-1 receptor, promote proliferation of large bowel cells, and induce carcinogenesis [21]. Second, lipid abnormalities might be associated with the production of bile acids, which have been proven to increase the risk of CRC [22]. It is reasonable to anticipate that serum lipids play a role in polyp recurrence. However, the relationship between serum lipid levels and polyp recurrence remains poorly understood.

In the current study, baseline lipid profiles as well as other baseline parameters were compared between patients with recurrent and non-recurrent polyps. The aim of this study was to evaluate the relationship between serum lipid profiles and polyp recurrence and identify potential markers that can be used to predict polyp recurrence.

## Materials and methods

### Participants

The participants of this retrospective study were selected from patients in the Endoscopic Center of the Gastroenterology Department at Meizhou People’s Hospital who underwent complete colonoscopy and first-time removal of colorectal polyps from January 2018 to June 2019. The inclusion criteria were a histological diagnosis of colorectal polyps, at least one follow-up colonoscopy performed at more than 6 months later after polyp removal, and sufficient baseline clinical and laboratory data. Patients were excluded if they had present/previous gastrointestinal tumors or cardiac or pulmonary disease. The study was approved by the Ethics Committee of Meizhou People’s Hospital Affiliated to Sun Yat-Sen University.

**Fig. 1:**
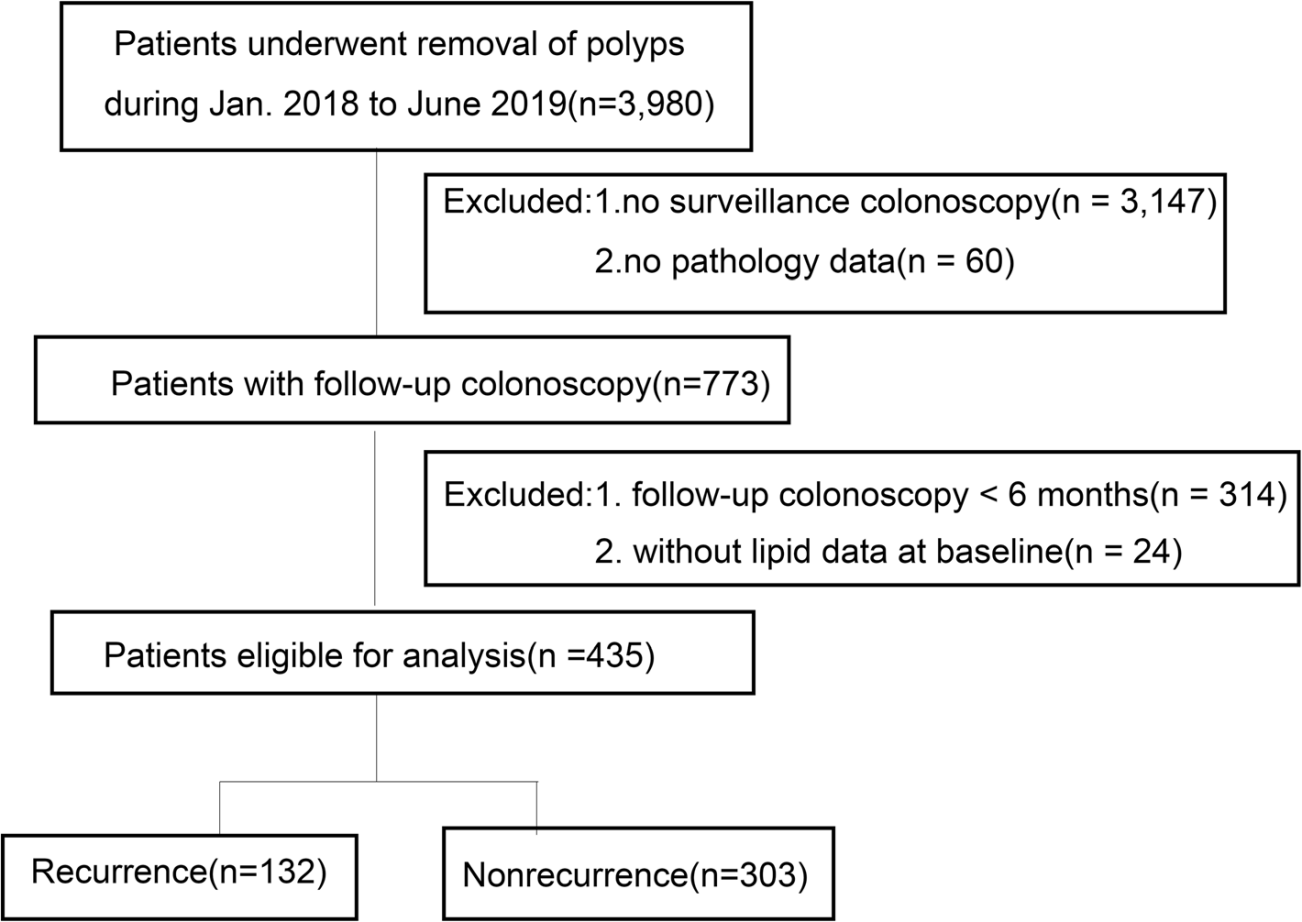
Inclusion and exclusion criteria of the study participants

A total of 3980 patients underwent an initial colonoscopy examination and removal of colorectal polyps during the study period. Of these 3980 patients, 3207 were excluded because they did not undergo follow-up colonoscopy. Of the remaining 773 patients, 314 lacked follow-up colonoscopy data > 6 months after removal and 24 lacked baseline lipid data. Finally, 435 patients were included in the analysis (Fig. 1).

### Colonoscopy procedure

Patients were required to consume a liquid diet for 24 h before the examination, and polyethylene glycol was used for standard bowel preparation. A complete colonoscopy including a good bowel preparation, colonoscopy reaching the cecum, and removing visualized lesions by endoscopic mucosal resection (EMR) was performed by experienced physicians. Biopsy specimens were inspected under a microscope by experienced pathologists.

### Polyp classification and definition of polyp recurrence

The pathologic characteristics of the colorectal polyps were obtained from the patients’ pathology reports. The polyps were classified into hyperplastic polyps, inflammatory polyps, tubular adenoma, or tubulovillous adenoma. Advanced polyps were diagnosed if one or more of the following conditions were met: tubular adenoma of ≥10 mm in diameter, tubulovillous adenoma, or the presence of high-grade dysplasia [23]. The location of the polyps was defined as proximal (cecum, ascending colon, hepatic flexure, transverse colon, and splenic flexure) or distal (descending colon, sigmoid colon, and rectum). In patients with multiple polyps, the histologic type, size, and location was based on the largest and/or most advanced adenoma.

Polyp recurrence was defined as the discovery of any polyp in the follow-up colonoscopy examination performed at least 6 months after the initial removal, whether at the same location or at other locations [12, 24].

### Lipid profile testing and clinical data collection

Peripheral venous blood samples were collected within 24 h after admission. The fasting lipid profiles, including the levels of total cholesterol, TG, high-density lipoprotein cholesterol (HDL-C), and LDL-C, were examined by selective solubilization (AU5800 analyzer; Beckman Coulter, Brea, CA, USA). The apolipoprotein A1 (ApoA1) and apolipoprotein B (ApoB) levels were tested using standard turbidimetric immunoassays (AU5800 analyzer; Beckman Coulter). Baseline characteristics including sex, age, BMI, drinking/smoking history, hypertension, and diabetes mellitus were collected from the medical records.

Drinking was defined as positive when alcohol consumption amounted to > 30 g/day. Hypertension was defined as blood pressure of ≥130/85 mmHg and/or current use of antihypertensive medication. Diabetes mellitus was diagnosed if the patient had a fasting blood glucose level of ≥126 mg/dL, a random glucose level of ≥200 mg/dL, or was taking an antidiabetic medication [25]. Dyslipidemia was defined as a total cholesterol level of ≥5.17 mmol/L, TG level of ≥1.7 mmol/L, HDL-C level of < 1.04 mmol/L, LDL-C level of ≥4.14 mmol/L, or current treatment with antidyslipidemic medication.

### Statistical analysis

Statistical analyses were performed using SPSS software version 22.0 (IBM Corp., Armonk, NY, USA). Continuous variables are expressed as mean ± standard deviation, and categorical variables are expressed as proportion. The normality of the distribution of continuous variables was evaluated with the Kolmogorov–Smirnov test. Continuous variables were tested by Student’s t test, whereas categorical variables were analyzed by the chi-square (χ^2^) test or Fisher’s exact test. Bonferroni correction was used for multiple comparisons. Logistic regression analysis was used to investigate the association of serum lipids and polyp recurrence. Odds ratios (ORs) were calculated by adjusting for variables that were distributed differently between patients with and without recurrence. All tests were two-sided, and a *P* value of < 0.05 was considered statistically significant.

## Results

### Patients’ baseline characteristics

In total, 435 patients were analyzed in the present study. Each patient completed a follow-up of at least 1.5 years unless found to have recurrent polyps by colonoscopy. The baseline characteristics of the 435 patients are presented in Table 1. The patients’ mean age was 56.6 ± 10.7 years. Male patients (56.3%) slightly outnumbered female patients. Few patients had a drinking habit (2.8%) or smoking habit (11.0%). The proportions of patients with hypertension, diabetes mellitus, and dyslipidemia were 19.1, 6.9, and 26.9%, respectively.

**Table 1:** Baseline characteristics of the study participants

Parameter	Total (*n* = 435)	Recurrent (*n* = 132)	Nonrecurrent (*n* = 303)	*P-*value
Age (years)	56.6 ± 10.7	58.6 ± 9.7	55.7 ± 10.9	0.008
Male (n, %)	245(56.3%)	92(69.7%)	153(50.5%)	< 0.001
Follow-up (month)	10.8 ± 4.5	11.7 ± 5.0	10.4 ± 4.2	0.003
BMI (kg/m^2^)	23.2 ± 3.3	22.9 ± 3.2	24.0 ± 3.5	0.003
Drink (n, %)	12(2.8%)	8(6.1%)	4(1.32%)	0.009
Smoke (n, %)	48(11.0%)	18(13.6%)	30(9.9%)	0.249
Hypertension (n, %)	83(19.1%)	29(22.0%)	54(17.2%)	0.353
Diabetes Mellitus (n, %)	30(6.9%)	9(6.8%)	21(6.9%)	1.00
Dyslipidemia (n, %)	117(26.9%)	42(31.8%)	75(24.8%)	0.127
TC (mmol/L)	5.49 ± 1.22	5.44 ± 1.23	5.40 ± 1.14	0.727
TG (mmol/L)	1.72 ± 1.15	1.74 ± 1.24	1.44 ± 0.92	0.006
HDL-C (mmol/L)	1.35 ± 0.33	1.32 ± 0.33	1.42 ± 0.38	0.008
LDL-C (mmol/L)	3.21 ± 0.85	3.12 ± 0.85	3.08 ± 0.83	0.646
ApoA1(g/L)	1.29 ± 0.33	1.28 ± 0.33	1.37 ± 0.40	0.033
ApoB(g/L)	1.02 ± 0.28	0.97 ± 0.28	0.96 ± 0.28	0.653
TG/HDL	1.47 ± 1.24	1.48 ± 1.37	1.16 ± 1.00	0.006

*BMI* body mass index, *TC* total cholesterol, *TG* triglycerides, *HDL-C* high-density lipoprotein cholesterol, *LDL-C* low-density lipoprotein cholesterol, *ApoA1* apoliprotein A1, *ApoB* apoliprotein B

*P*-value: Comparison between recurrent and nonrecurrent performed by Student’s t test or Chi-square (χ^2^) test

During the 1.5-year follow-up, polyp recurrence was found in 132 patients (30.3%). Patients with recurrent polyps were older than those without recurrent polyps (*P* <  0.008) and comprised a higher proportion of male patients (*P* <  0.001). Patients with recurrent polyps had a higher TG level (*P* = 0.006) and lower HDL-C and ApoA1 levels (*P* = 0.008 and 0.033, respectively). The TG to HDL ratio (TG/HDL) was significantly higher in patients with than without recurrent polyps (*P* = 0.006).

### Comparison of polyp characteristics between patients with and without recurrence

The characteristics of the removed polyps are presented in Table 2. Most polyps (74.94%) had a diameter of ≤10 mm. Large polyps (≥ 20 mm) were significantly more frequent in patients with than without recurrence (16.67% vs. 8.25%, respectively; *P* = 0.036). The number of polyps was significantly different between patients with and without recurrence (*P* <  0.001). Patients with recurrence had a significantly higher proportion of multiple polyps (≥ 3) than those without recurrence (66.67% vs. 34.98%, respectively; *P* <  0.001). The histology of the polyps was different between patients with and without recurrence (*P* = 0.028), with a higher rate of tubulovillous adenoma found in patients with than without recurrence (21.97% vs. 11.55%, respectively; *P* = 0.02). The polyp locations and adenoma classifications were not significantly different between patients with and without recurrence.

**Table 2:** Comparison of colorectal polyps between patients with and without polyp recurrence

Parameter	Total (*n* = 435)	Recurrent (*n* = 132)	Nonrecurrent (*n* = 303)	*P* *-*value	*P-*value^*#*^
Polyp size (mm)				0.019	
≤ 10	326 (74.94%)	96(72.73%)	230(75.91%)	0.482	
10–19	62 (14.25%)	14(10.61%)	48(15.84%)	0.151	
≥ 20	47 (10.80%)	22(16.67%)	25(8.25%)	0.012	0.036
Polyp number, n (%)				< 0.001	
1	139(31.95%)	20(15.15%)	119(39.27%)	< 0.001	< 0.001
2	101(23.22%)	24(18.18%)	77(25.41%)	0.097	
≥ 3	194(44.60%)	88(66.67%)	106(34.98%)	< 0.001	< 0.001
Polyp type, n (%)				0.028	
Hyperplastic	53(12.18%)	17(12.88%)	36(11.88%)	0.770	
Inflammatory	33(7.59%)	7(5.30%)	26(8.58%)	0.425	
Tubular adenoma	285(62.52%)	79(59.85%)	206(67.99%)	0.101	
Tubulovillous adenoma	64(14.71%)	29(21.97%)	35(11.55%)	0.005	0.02
Polyp location, n (%)				0.149	
Proximal	137(31.50%)	48(36.36%)	89(29.37%)		
Dismal	298(68.50%)	84(63.64%)	214(70.63%)		
Adenoma, n (%)				0.116	
Advanced	150(42.86%)	53(49.07%)	97(40.08%)		
Nonadvanced	200(57.14%)	55(50.93%)	145(59.92%)		

*P*-value: Comparison between recurrent and nonrecurrent performed by Chi-square χ^2^ test

*P*-value^#^: *P* value after Bonferroni correction for multiple comparisons

### Comparison of serum lipid profiles between patients with and without polyp recurrence

The serum lipid levels were analyzed in patients with different colorectal polyps. Among patients with hyperplastic polyps, those with recurrence had a higher TG level and TG/HDL (*P* <  0.05). Among patients with tubulovillous adenomas, those with recurrence had a higher TG level and TG/HDL (*P* <  0.05) and lower HDL and ApoA1 levels (*P* <  0.05). Among patients with inflammatory polyps or tubular adenomas, those with recurrence had a higher TG level and TG/HDL; however, the difference did not reach statistical significance. The LDL-C level tended to be high in all patient groups, but the differences were not statistically significant among the four polyp groups (Table 3).

**Table 3:** Comparison of serum lipid and lipoproteins between patients with and without polyp recurrence

	Hyperplastic polyp(*n* = 53)		Inflammatory polyp(*n* = 33)		Tubular adenoma(*n* = 285)		Tubulovillous adenoma(*n* = 64)	
	Recurrent	Nonrecurrent	Recurrent	Nonrecurrent	Recurrent	Nonrecurrent	Recurrent	Nonrecurrent
TG (mmol/L)	2.04 ± 1.48	1.29 ± 0.71*	1.98 ± 1.84	1.44 ± 0.85	1.60 ± 1.04	1.50 ± 0.97	1.91 ± 1.44	1.24 ± 0.82*
CHO (mmol/L)	5.64 ± 1.19	5.52 ± 1.53	5.23 ± 0.81	4.85 ± 1.26	5.31 ± 1.18	5.39 ± 1.05	5.73 ± 1.44	5.72 ± 0.98
HDL-C (mmol/L)	1.28 ± 0.35	1.45 ± 0.34	1.30 ± 0.34	1.42 ± 0.42	1.36 ± 0.33	1.40 ± 0.36	1.26 ± 0.32	1.50 ± 0.48*
LDL-C (mmol/L)	3.20 ± 0.89	3.10 ± 1.02	3.00 ± 0.61	2.66 ± 0.87	3.06 ± 0.81	3.10 ± 0.79	3.29 ± 0.98	3.26 ± 0.72
ApoA1 (g/L)	1.24 ± 0.47	1.41 ± 0.39	1.33 ± 0.42	1.32 ± 0.29	1.31 ± 0.29	1.35 ± 0.41	1.23 ± 0.32	1.45 ± 0.36*
ApoB (g/L)	1.00 ± 0.31	0.96 ± 0.31	1.00 ± 0.28	0.80 ± 0.25	0.93 ± 0.24	0.97 ± 0.27	1.05 ± 0.34	0.97 ± 0.25
TG/HDL	1.74 ± 1.64	0.96 ± 0.74*	1.75 ± 1.90	1.16 ± 0.93	1.36 ± 1.19	1.23 ± 1.07	1.60 ± 1.55	0.93 ± 0.81*

*: *P* < 0.05, comparison between recurrent and nonrecurrent performed by Student’s t test

### Association of elevated TG level with recurrence of colorectal polyps

Regression logistic analysis was used to determine the risk factors associated with polyp recurrence. As shown in Table 4, among patients with overall polyps, male sex [OR, 1.75; 95% confidence interval (CI), 1.06–2.89], the number of polyps (OR, 3.42; 95% CI, 2.15–5.45) and the BMI (OR, 1.11; 95% CI, 1.02–1.19) significantly increased the risk of polyp recurrence. Among patients with advanced adenoma, the number of polyps (OR, 3.28; 95% CI, 1.44–7.47), the polyp size (OR, 2.86; 95% CI, 1.24–6.61), and the elevated TG level (OR, 1.55; 95% CI, 1.02–2.35) remained significant predictors of polyp recurrence.

**Table 4:** Multivariate analysis of risk factors associated with polyp recurrence

Parameter	All polyps(***n*** = 435)		Advanced adenomas(***n*** = 150)	
	OR (95% CI)	*P*-value	OR (95% CI)	*P*-value
Age (years)	1.02(1.00–1.04)	0.063	1.02(0.99–1.06)	0.231
Male	1.75(1.06–2.89)	0.029	0.94(0.41–2.16)	0.891
BMI (≥25 kg/m^2^)	1.11(1.02–1.19)	0.012	0.96(0.84–1.09)	0.501
Number (≥ 3)	3.42(2.15–5.45)	< 0.001	3.28(1.44–7.47)	0.005
Size (≥ 20 mm)	1.72(0.87–3.40)	0.117	2.86(1.24–6.61)	0.014
TG (≥ 1.7 mmol/L)	1.15(0.90–1.48)	0.257	1.55(1.02–2.35)	0.039
HDL-C (≤1.04 mmol/L)	1.47(0.50–4.31)	0.481	0.73(0.11–4.70)	0.737
ApoA1 (<1.0 g/L)	0.62(0.25–1.58)	0.318	0.364(0.06–2.40)	0.294

*OR* odd ratio, *CI* confidence interval, *BMI* body mass index, *TG* triglyceride, *HDL-C* high-density lipoprotein cholesterol, *ApoA1* apoliprotein A1

## Discussion

Recurrence of colorectal polyps is common and remains a crucial problem in the prevention of CRC. Although several factors have been revealed to impact the recurrence rate, few studies have focused on the role of the lipid profile. The present study analyzed the baseline lipid profiles of patients with colorectal polyps and the recurrence of polyps with a modest follow-up duration. The data showed that patients with recurrence had a higher TG level and lower HDL-C and ApoA1 levels than those without recurrence. To the best of our knowledge, this is the first study to demonstrate that an elevated TG level significantly predicts polyp recurrence in patients with advanced adenoma.

Colorectal polyps are precursors of CRC, and removal of polyps effectively reduces the risk of developing CRC. Endoscopic resection is now widely used to excise colorectal polyps and thus reduces the incidence of death caused by CRC [3]. However, the high recurrence rate after piecemeal resection poses a serious clinical problem. One clinical trial showed that 20 to 50% of patients with colorectal adenomas developed recurrence within a 3- to 5-year period [26]. During the short-term follow-up in the present study, we observed polyp recurrence in 132 of 435 patients (30.34%). Elucidation of the causative factors of polyp recurrence may practically reduce or delay the recurrence of polyps.

Several factors are reportedly associated with polyp recurrence, including age, sex, lifestyle, genetic background, polyp characteristics, and procedural factors [9, 27–30]. The present study compared the baseline clinical characteristics of both the patients and the colorectal polyps. In accordance with previous studies, significant differences were observed in age, sex, polyp size, and polyp number between patients with and without polyp recurrence. The proportion of people with obesity in the general population has sharply increased, and obesity causes or aggravates disease. Epidemiologic studies have suggested that obesity is a risk factor for colorectal adenomas [31] and CRC mortality [32]. Several studies have determined that obese individuals (or those with a high BMI) have a higher risk of recurrence than non-obese individuals; the underlying mechanism is considered to be the fact that obesity increases the insulin and IGF-1 levels, pushing non-advanced colorectal polyps into the advanced stage and causing recurrence [12, 33, 34]. Consistent with previous findings, we observed that patients with recurrent polyps had a higher BMI, suggesting that the BMI is an independent risk factor for polyp recurrence.

Several studies have investigated the influence of lipid profiles on the development of colorectal polyps. Most of these studies suggested that a higher serum TG level was significantly associated with an increased risk of colorectal adenomas, while a higher HDL level was inversely associated with the development of adenomas [35–39]. Obesity causes oxidative stress and inflammation, resulting in lipid accumulation within organs, which is known to cause insulin resistance and hyperglycemia. Hepatic insulin resistance results in increased TG synthesis and gluconeogenesis, leading to serum hypertriglyceridemia, hypertension, and hyperglycemia [40]. In the current study, we compared the baseline lipid profiles between patients with and without recurrent polyps. The data showed that patients with recurrence had significantly higher TG levels and lower HDL-C and ApoA1 levels. The multivariate logistic regression analysis suggested that an elevated TG level was an independent risk factor for polyp recurrence in patients with advanced adenomas. However, the molecular mechanism by which lipids regulate polyp recurrence remains poorly understood.

Two possible mechanisms may contribute to this phenomenon. First, hypertriglyceridemia is associated with hyperinsulinemia and insulin resistance, which drive cell proliferation via receptors on normal cells and cancer cells of the large bowel [41]. Second, hypertriglyceridemia induces the release of more inflammatory cytokines such as interleukin 6 and tumor necrosis factor alpha and reduces the release of anti-inflammatory cytokines such as interleukin 10 [42, 43], which may create a cellular environment conducive to neoplasia. Studies have suggested that cancer stem cells (CSCs) are responsible for cancer recurrence [44, 45]. Recent studies have shown that lipid droplet-enriched CSCs had higher tumorigenic potential and that lipid droplet accumulation in CSCs contributed to polyp development [46, 47]. We speculate that TG influences polyp recurrence through CSCs by perhaps increasing their proliferation capability or stimulating signaling pathways that promote cell invasion. Further experimental evidence is needed to prove this theory.

### Study strengths and limitations

Researchers have largely focused on risk factors for colorectal polyp recurrence and have identified some baseline colonoscopy features. However, whether lipid abnormalities contribute to colorectal polyp recurrence remains poorly understood. The present study suggests that an elevated TG level is an independent risk factor for recurrence in patients with advanced adenoma, which can be regarded as the main strength of the present study.

This study also has some limitations. First, the retrospective design of the study made it impossible to confirm the accuracy of the self-reported data, such as the BMI and drinking and smoking habits, therefore causing possibly misleading results. Second, the 1.5-year follow-up may not have been long enough to observe all instances of recurrence, which may be another confounding factor in the data analysis. Third, the molecular mechanism remains unclear, and experimental evidence is needed to strengthen the link between an elevated TG level and polyp recurrence.

## Conclusions

The present study investigated the correlation between lipid profiles and recurrence of colorectal polyps. After adjusting for age, sex, BMI, and polyp number and size, the multivariate analysis suggested that an elevated TG level was an independent risk factor for polyp recurrence in patients with advanced adenomas. Because the serum TG level is extensively examined in clinical practice, it may serve as practical marker for defining patients at high risk of polyp recurrence, and helps to protect against CRC.

## Data Availability

The datasets used and analyzed during the current study will be provided by the corresponding author upon reasonable request.

## Abbreviations

CRC: Colorectal cancer
WHO: World Health Organization
EMR: Endoscopic mucosal resection
TC: Total cholesterol
TG: Triglycerides
HDL-C: High-density lipoprotein cholesterol
LDL-C: Low-density lipoprotein cholesterol
ApoA1: Apoliprotein A1
ApoB: Apoliprotein B
BMI: Body mass index
DM: Diabetes mellitus
OR: Odd ratio
CI: Confidence index
